# Effect of Different Root Canal Irrigant Solutions on the Release of Dentin-Growth Factors: A Systematic Review and Meta-Analysis

**DOI:** 10.3390/ma14195829

**Published:** 2021-10-05

**Authors:** Sandro Tavares, Andrea Pintor, Carlos Fernando de Almeida Barros Mourão, Marcela Magno, Pietro Montemezzi, Roberto Sacco, Gutemberg Alves, Miriam Zaccaro Scelza

**Affiliations:** 1 Post-Graduate Program in Dentistry, Universidade Federal Fluminense, Niterói 24020-140, RJ, Brazil; sandro.tavares04@gmail.com (S.T.); roberto.sacco@manchester.ac.uk (R.S.); 2 Department of Pediatric Dentistry and Orthodontics, Universidade Federal do Rio de Janeiro, Rio de Janeiro 21941-902, RJ, Brazil; andrea_pintor@hotmail.com (A.P.); marcela.magno@hotmail.com (M.M.); 3 Clinical Research Unit of the Antonio Pedro Hospital, Universidade Federal Fluminense, Niterói 24033-900, RJ, Brazil; m.montemezzi@libero.it (P.M.); gutopepe@yahoo.com.br (G.A.); 4 Division of Dentistry, School of Medical Sciences, University of Manchester, Manchester M13 9PL, UK; 5 Laboratory of Experimental Culture Cell (LECCel), Department of Endodontics, Faculty of Dentistry, Universidade Federal Fluminense, Niterói 24020-140, RJ, Brazil

**Keywords:** dentin, growth factor, root canal irrigants

## Abstract

Irrigant solutions are used to promote dentin-growth factors (GF) release for regenerative endodontics. This review aimed to evaluate the reports comparing the release of GFs using different root canal irrigant solutions. Eligible studies compared the in vitro GF release in human teeth after the use of at least two distinct solutions. A search was conducted on Pubmed, Scopus, Web of Science, and Lilacs on 11 August 2021. Risk of bias was assessed using SciRAP. Study characteristics and quantitative data were extracted, and meta-analyses were performed for the mean difference (95% confidence interval) of the release of transforming growth factors Beta 1 (TGF-β1) by EDTA compared to other irrigants. Of sixteen eligible studies, eight were included in quantitative analysis. ELISA assays showed higher TGF-β1 release from 10% EDTA compared to 10% citric acid (*p* < 0.00001). Immunogold assays showed higher levels of TGF-β1 for 17% EDTA (*p* < 0.00001) compared to 10% citric acid. GRADE identified a low to very low certainty of evidence. These results point to an increased release of TGF-β1 in dentin treated with EDTA. The high heterogeneity and very low certainty of the evidence demand further studies before EDTA indication as a better irrigant for regenerative endodontics. Registration: CRD42020160871 (PROSPERO).

## 1. Introduction

Endodontic therapy remains a challenge for immature permanent teeth since the cessation of root development can make the tooth more fragile, increasing root fracture rates in the medium- and long-term [1]. In this sense, regenerative endodontics stands out by aiming to replace damaged dental structures through biological procedures, including the participation of cells of the dentin–pulp complex [2]. It is noteworthy that the application of regenerative procedures does not exclusively address immature teeth, since a recent systematic review with meta-analysis shows a high success rate of its application also in mature permanent teeth [3].

Regenerative endodontic therapies are often based on the principles of tissue engineering, grounded on the triad: stem cells, biomimetic scaffolds, and bioactive growth factors [4]. Stem cells derived from teeth act in the maintenance of pulp homeostasis, in addition to having high plasticity and pluripotency, being able to differentiate into odontoblast-like cells [5]. In this sense, it is noteworthy that the apical papilla region is full of mesenchymal stem cells (SCAPs), which can survive endodontic infection, and jointly with the Hertwig’s epithelial root sheath, they direct root development. Therefore, regenerative endodontic therapies are necessary for immature teeth to maintain the viability of these structures and ensure the complete root maturation process [6,7]. In this sense, a favorable microenvironment is required for the tissue neoformation process to occur during regenerative therapy, allowing the interaction of stem cells with the biomimetic scaffolds [3] on a process modulated by growth factors. Consequently, growth factors play an essential role as biological inductors [4]. 

These bioactive signaling molecules modulate cellular behavior, including migration, proliferation, differentiation, and apoptosis, with well-known effects on cells from the dentin–pulp complex [8]. The dentin matrix is an extensive reservoir of growth factors, including the transforming growth factors beta 1, 2, and 3 (TGF-β1, TGF-β2, and TGF-β3), platelet-derived growth factor (PDGF), bone morphogenetic proteins 2, 4, and 7 (BMP-2, BMP-4, and BMP-7), insulin-like growth factors 1 and 2 (IGF-I and IGF-II), hepatocyte growth factor (HGF), vascular endothelial growth factor (VEGF), and adrenomedullin (ADM) [9]. These molecules are expressed in their inactive forms in the extracellular space and quickly bind to the extracellular matrix (ECM) components during dentinogenesis, preventing proteolytic degradation [10]. 

Growth factors entrapped in dentin can be reactivated and released through its demineralization, due to the action of acidogenic byproducts of caries [11,12]. Similarly, agents such as organic acids, endodontic irrigants, and demineralizing solutions contribute to releasing growth factors bound to the dentinal matrix [13]. The release gradient of these bioactive molecules is a possible explanation for dentin’s repairing response, considering that they act as chemotactic agents, stimulate the secretory activity of preexisting odontoblasts, and provide effects on the differentiation of pulp stem cells from osteodentin [14]. This phenomenon has instigated both researchers and endodontists to consider using root canal irrigants solutions on regenerative procedures to conduct the tooth sensitivity recovery/induction [15]. 

In a context where the interrelationship of the elements of the bioengineering triad is fundamental for the effectiveness of regenerative endodontics, and where the regenerative potential can be optimized with increased release of growth factors on a dose-dependent pattern, it becomes clinically relevant to know which root canal irrigant solutions favor the release of these biomolecules trapped in dentin. However, regenerative endodontic therapy is still evolving, lacking in evidence from the translational and clinical research to support the clinician’s informed choice for the best therapeutic irrigating protocol to promote the release of dentin-growth factors and tissue repair. Furthermore, clinical studies’ inherent heterogeneity and complexity have impaired the assessment of data regarding the release of growth factors during regenerative endodontic therapies. As a result, most data have been obtained through in vitro and ex vivo settings, where experimental issues can be isolated and controlled. Nevertheless, data generated in laboratory research may be relevant to the design of clinical trials when they present the translational potential to improve clinical practice. Understanding these studies’ scopes and findings is of great importance to an evidence-based approach to regenerative endodontic future clinical research and current practices.

Therefore, this systematic review aimed to evaluate the reports comparing the release of dentin-growth factors through the action of different endodontic irrigants solutions and assess the level of evidence available in the updated scientific literature, intending to answer the PICO-focused question: “Does the choice of root canal irrigant solution influence the release of dentin-growth factors in human teeth?”

## 2. Materials and Methods

The systematic review’s protocol was registered in PROSPERO (CRD42020160871) (University of York, York, UK) and reported following the PRISMA (Preferred Reporting Items for Systematic reviews and Meta-Analyses) guideline principles [16].

### 2.1. Eligibility Criteria

The eligibility criteria were based on the PICOS strategy (P: population, I: intervention, C: comparison, O: outcome, S: study design), considering (P) = human teeth; (I) = use of a root canal endodontic irrigant; (C) = use of distinct root canal endodontic irrigants; (O) = release of dentin-growth factors; and study design (s) = in vitro studies. 

The included in vitro studies evaluated the release of dentin-growth factors in human teeth after using at least two distinct root canal endodontic irrigants. Conversely, studies conducted on nonhuman teeth or that evaluated the release of dentin-growth factors in human teeth without comparing at least two different endodontic irrigants were excluded. In addition, the literature reviews, guidelines, book chapters, editorials, opinions, conferences, and other non-experimental publications were excluded. 

The studies were grouped for data synthesis according to the GFs release evaluation methods, enzyme-linked immunosorbent assay (ELISA) and immunogold assay.

### 2.2. Information Sources

A broad search was performed on 11 August 2021, in the electronic databases: Pubmed, Scopus, Web of Science, and Virtual Health Library (LILACS). The gray literature was consulted on Opensigle (http://www.opengrey.eu/, accessed on 11 August 2021) (EAGLE-European Association for Grey Literature Exploitation, Brussels, Belgium), and a manual search in the references of the included articles was also performed. 

### 2.3. Search Strategy

The MeSH (medical subject headings) terms, synonyms, related terms, and free terms were included in the search strategies. The terms used in the search key referring to root canal irrigants solutions were taken from a previous review study that cites the main endodontic irrigants [9,17]. The terms used in the growth factor search key were initially taken from [9]. The terms were combined to refine the search results using the search keys presented in Supplementary Materials Table S1 available at Mendeley Data (https://data.mendeley.com/datasets/s8znfrtjxx/1) (accessed on 21 September 2021)**.**

### 2.4. Selection Process

The identified documents were exported to Mendeley Desktop software (Mendeley Ltd., London, UK) and organized, and duplicates were removed automatically but also manually checked. Two examiners (SOT and MZS) screened titles and abstracts of all entries in consensus meetings, so that only studies that matched the eligibility criteria were selected. If in doubt, the full article was read. Subsequently, the potentially eligible studies were reevaluated in full text by the two reviewers (SOT and MZS). Disagreements were solved by a third reviewer (GA) during the consensus meetings. 

### 2.5. Data Collection Process and Data Items

Data collection from the included articles was performed by two independent reviewers (SOT and MZS). The main characteristics of included studies were organized according to 1-the author and year of publication; 2-the sample type; 3-root canal irrigants used; 4-growth factor analyzed; 5-evaluation method; and 6-main conclusions.

For the analysis of the growth factor release by endodontic irrigants, all quantitative data were extracted from the studies included through the means and standard deviations as well as exposure time, assessment time, and sample size. The measurement units of the studies with ELISA evaluation were established pg/mL and converted to this unit whenever necessary and possible. For the studies that used immunogold assays, data were represented as were mean particle numbers. For studies that did not provide the numerical value in full and presented only graphics, the CorelDRAW Graphics Suite 2018 software (Corel Corporation, Ottawa, ON, Canada) was used to extract means and standard deviations from the graphical data. In addition, when necessary, medians and interquartile were converted to mean and SD [18] using a calculator available at http://vassarstats.net/median_range.html (accessed on 16 August 2021).

### 2.6. Studies Risk of Bias Assessment

The risk of bias assessment at the methodological and reporting levels was conducted by two independent reviewers (SOT and MZS), according to the in vitro reporting checklist from the Science in Risk Assessment and Policy-SciRAP (www.scirap.org, accessed on 14 August 2021) (Stockholm University, Stockholm, Sweden, and Karolinska Institute, Solna, Sweden). The SciRAP assesses three domains. The first is the reporting quality, which refers to the report of the included study related to test compounds and controls, test system, dosing and administration, data collection and analysis, funding sources, and competing interests. The domain named methodological quality assesses the criteria on study design, methods, and management that could influence outcomes. Finally, it also provides an assessment of the study’s relevance concerning the review question. While this instrument was designed initially for toxicological studies [19], its domains could be easily interpreted to evaluate other types of in vitro studies. These domains generate a score from 0 to 100, in which the authors established, by consensus, a cut-off point of 70 as the minimum accepted quality assessment score. The obtained scores for reporting and methodological quality were registered and compared to a 70 cut-off point, aiming to assess the risk of bias in the included studies. Studies rated with reporting and methodological quality scores higher than 70 points were considered with good reporting quality and methodological quality. Similarly, the cut-off point of 70 was used to assess the risk of bias between the studies since the SciRAP tool does not dictate values for reporting or methodological quality categorization. In order to assess the risk of bias between the included studies, the results of the individual analyses were summarized and compared on an overall qualitative synthesis regarding each of the three domains. 

### 2.7. Synthesis Methods and Effect Measures

The data from studies were analyzed using the RevMan software (Review Manager v. 5.3, the Cochrane Collaboration; Copenhagen, Denmark) to perform a quantitative assessment of the available evidence. Since all studies employed EDTA as a comparator and included TGF-β1 as one of the assessed analytes, the existing data allowed one to evaluate the influence of different irrigants solution, compared to EDTA, on radicular dentin TGF-β1 release levels. Ten analyses were performed to evaluate comparisons with two or more studies: (i) 10% EDTA versus 10% citric acid; (ii) 17% EDTA versus distilled water; (iii) 17% EDTA versus 10% EDTA; (iv) 17% EDTA versus 10% citric acid; (v) 17% EDTA versus citric acid phosphate buffer; (vi) 17% EDTA versus phytic acid; (vii) 17% EDTA versus 9% etidronic acid; (viii) 17% EDTA versus 1.5% NaOCl + 17% EDTA; (ix) 17% EDTA versus 2.5% NaOCl + 17% EDTA; and (x) 17% EDTA versus 17% EDTA + 0.008% Benzalkonium chloride (BAC). Only studies that assessed the growth factor through the ELISA method were included in these comparisons to reduce heterogeneity. Additional analyses were performed comparing the influence of EDTA (10% and 17%) versus 10% citric acid on the release of TGF-β1 levels evaluated through the immunogold method. 

Mean standard deviation (SD) and the total number of samples in each group (EDTA and other irrigants) were included, and the mean difference (MD) with a 95% confidence interval (CI) was calculated. A fixed-effect model was applied when a low number of studies was included (three or fewer comparisons), and a random effect model was applied when four or more comparisons were included in the meta-analysis [20]. Heterogeneity was tested using the I^2^ index. 

### 2.8. Certainty of Evidence Assessment

The certainty of the evidence (certainty in the estimates of effect) was determined for each outcome using the grading of recommendations assessment, development, and evaluation (GRADE) approach [21] (https://gradepro.org/, accessed on 20 September 2021) (GRADEpro GDT, 2020, GRADE working group, Hamilton, ON, Canada), with some adjustments for in vitro studies. Indirectness was evaluated considering the types of dentin samples (slices, powdered, and segments) and irrigants’ concentrations. The magnitude of effects was calculated as a fold change from the mean EDTA concentration. Ratios > 1.5 and <3 were considered large, and >3 were considered very large. As originally recommended, all in vitro (nonclinical, nonrandomized) evidence was classified to start as of low certainty. The level of evidence decreased to very low if “serious”, or “very serious” issues were identified regarding the risk of bias, imprecision, inconsistency, indirectness, and publication bias. The GRADE approach was performed for each meta-analysis comparison, and a quality certainty of the evidence for FGF, VEGF, BMP2, IGF-I, BMP-7, and overall growth factors were also performed.

## 3. Results

### 3.1. Study Selection

**Figure 1:**
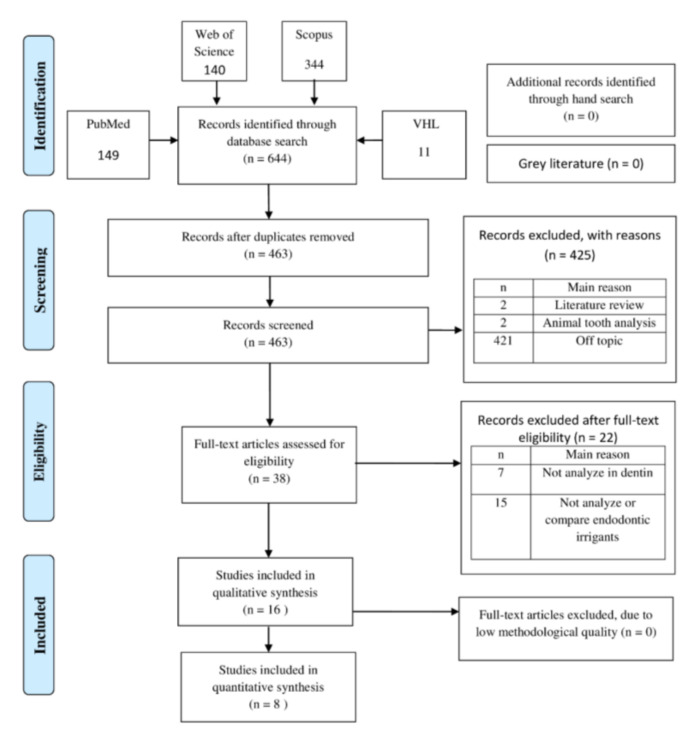
PRISMA flow diagram.

Figure 1 presents the study selection process, where a total of 644 documents were retrieved. After applying the eligibility criteria, 38 articles were read in full. Of these, 15 were excluded for not investigating or comparing endodontic irrigants, and seven studies did not include dentin. Sixteen studies that met the criteria were included in the qualitative synthesis [10,13,22,23,24,25,26,27,28,29,30,31,32,33,34,35], and eight were included in the quantitative synthesis [10,13,22,24,29,32,33,35].

### 3.2. Characteristics of the Included Studies

The main characteristics of the included studies are shown in Table 1. Most studies have employed dentin slices as the test system [10,22,23,26,27,28,29,31,32,35]. Five studies used root canal segments [13,23,24,25,33], and two studies used solubilized dentin powder [30,34]. 

Regarding the endodontic irrigating solutions used, all studies evaluated the effects of 10% or 17% EDTA on dentin. The second most used irrigant was citric acid, employed in seven studies [10,13,24,27,28,32,35]. However, other irrigants were also investigated, as described in Table 2. 

While almost all studies included TGF-β1 as an analyte in the growth factor evaluation, except for Ferreira et al. [25] who only assessed VEGF, some additionally evaluated FGF [27,33], VEGF [24,25,27,32], IGF-I [24], and the BMP [24,27,32]. Regarding the analysis method, only two articles used methods other than ELISA, analyzing the presence of proteins through the slot blot technique [28] or immunogold staining [35]. Sadaghiani et al. [32] used both methods, ELISA and immunogold staining.

The main conclusions of the included studies are shown in Table 2. Regarding the mean TGF-β1 release, EDTA induces a higher release [10,30,31,34,35] similar result for IGF-I release in only one study [24]. One study indicated that the association of sodium hypochlorite with EDTA showed higher release of TGF-β1 than EDTA alone [33]. Sadaghiani et al. [32] found better results with EDTA when the evaluation was performed with ELISA, while an increased release of growth factors was evidenced for calcium hydroxide by immunoassays. The raw data are available at Mendeley Data as Supplementary Materials Table S2: https://data.mendeley.com/datasets/s8znfrtjxx/1 (accessed on 21 September 2021). Three studies identified a higher significant release of TGF-β1 [13,27,28] and BMP-2 [32] by citric acid.

VEGF release was not detected or only in small concentration under the conditioning of any irrigant in two studies [25,27], with a similar pattern found for FGF [33]. Sadaghiani et al. [32] showed a greater release of VEGF by calcium hydroxide in the immunogold assay, while 10% citric acid was more effective as detected by the ELISA method. Khan et al. [23] showed better results for VEGF release by 9% etidronic acid in dentin cylinders when compared to 17% EDTA and 1% phytic acid. 

### 3.3. Risk of Bias in Studies

The quality assessment results are summarized in Table 3, and the raw data are available at Mendeley Data (https://data.mendeley.com/datasets/s8znfrtjxx/1) (accessed on 21 September 2021). Zhao et al. [35] obtained the lowest score on reporting quality (71.88), while Chae et al. [13] obtained the lowest score on methodological quality (78.57). However, all included studies obtained a score above the cut-off, considered good reporting quality, methodological quality, and directly relevant to the topic addressed. 

The lower scores of the three domains of analysis were found for the reporting quality. The main concerns are related to test compound and controls [10,13,24,25,26,27,28,30,31,32,33,34,35], where in most studies, it is difficult to trace the origin of the irrigating solutions used, as well as their purity and dilution vehicle. Consequently, it also affected the methodological quality in relation to the composition of the root canal irrigant solutions [10,13,24,25,26,27,28,30,31,32,33,34,35]. In fact, this was one of the only common factors of concern among studies in this methodological quality assessment.

On the other hand, most studies fully met the test system domain, specifying the parameters used in the management of test substances, for both reporting quality [23,24,25,26,29,30,31,32,33,35] and methodological quality [10,22,23,24,25,26,27,28,29,30,31,32,33,34,35], and also the domain related to the doses used and the number of replicates performed, named the administration of test compound, for reporting quality [10,22,23,25,26,28,29,30,33,34] and methodological quality [10,22,23,24,25,26,27,28,29,30,32,33,34]. Furthermore, all studies were classified as directly relevant and with an overall low risk.

### 3.4. Results of Synthesis, Meta-Analyses, and Certainty of Evidence

Eight studies were not included in the meta-analyses because they used quite different assessment techniques or did not report sufficient quantitative data to be compared [23,25,26,27,28,30,31,34]. Atesci et al. [27] were not included since the methodology assessed the total growth factor in dentin and not the level of growth factor released. Ivica et al. [28] was the only study that performed the slot blot technique for assessing the dentin-growth factors release. Although Ferreira et al. [25], Aksel et al. [26], Graham et al. [34], and Duncan et al. [30] used the same methodology, they did not investigate similar irrigation solutions to allow a comparison, while Khan et al. [23] did not assess the TGF-β1 release. 

#### 3.4.1. 10% EDTA Versus 10% Citric Acid

**Figure 2:**
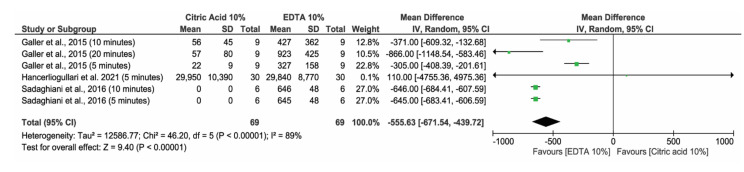
Forest plot for comparisons of TGF-β1 detected by ELISA method after exposure to 10% EDTA versus 10% citric acid.

Three studies [10,24,29] and six comparisons of means and standard deviation of TGF-β1 detected by ELISA method after exposure to 10% EDTA versus 10% citric acid, in distinct exposure times, were included in this analysis (Figure 2). In total, 138 samples were evaluated, and the mean difference of TGF-β1 released levels were higher for the 10% EDTA groups compared to 10% citric acid groups (MD −555.63 [−671.54, −439.72] *p* < 0.00001), however with considerable heterogeneity (I^2^ = 89%) and very low certainty of evidence according to GRADE (available at Mendeley Data as Supplementary Materials Table S3: https://data.mendeley.com/datasets/s8znfrtjxx/1) (accessed on 21 September 2021).

#### 3.4.2. 17% EDTA Versus Other Irrigants

**Figure 3:**
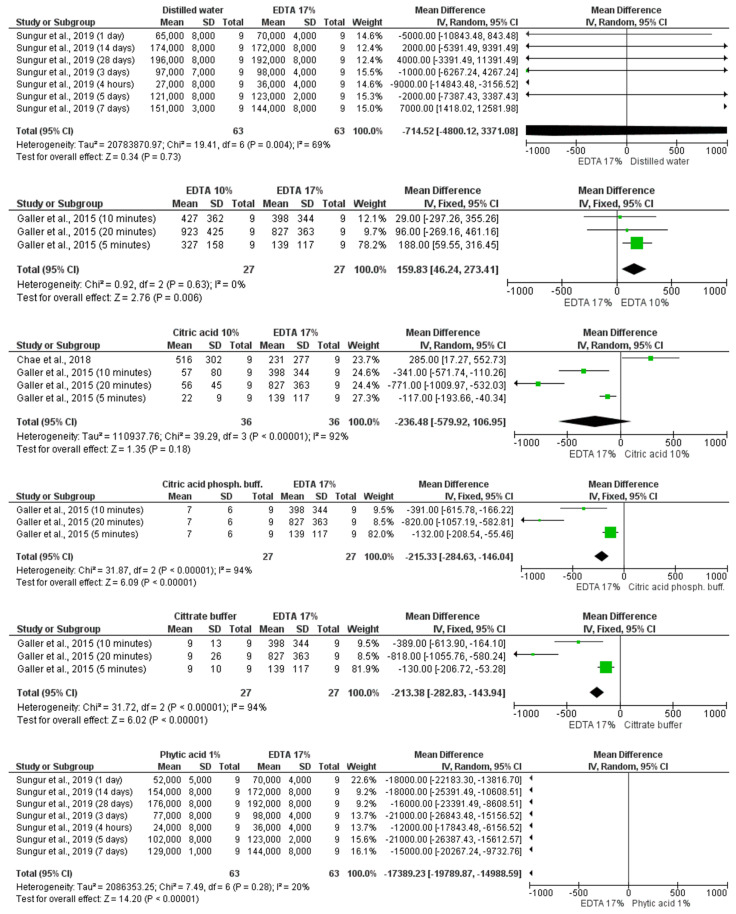

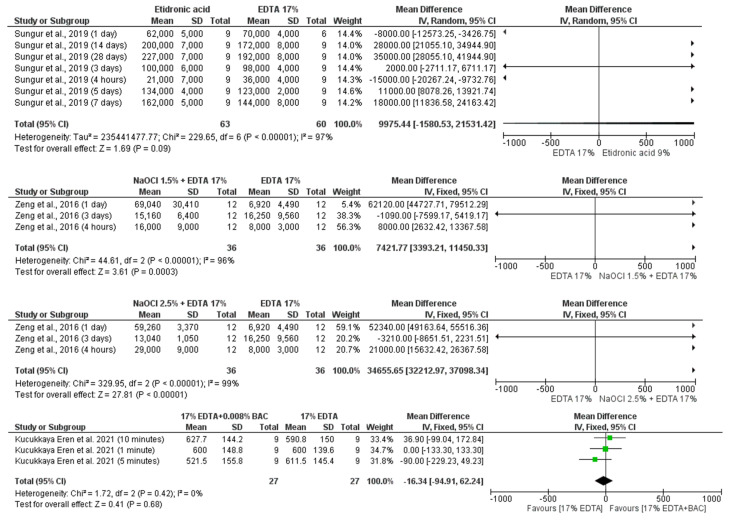
Forest plot for comparisons of TGF-β1 detected by ELISA method after exposure to 17% EDTA versus other irrigants.

Five studies [10,13,22,29,33] and 43 comparisons of means and standard deviation of TGF-β1 detected by ELISA method after exposure to 17% EDTA versus other irrigants were included in this analysis (Figure 3). Samples irrigated with 17% EDTA presented similar TGF-β1 release when compared to samples irrigated with distilled water (MD −714.52 [−4800.12, 3371.08] *p* = 0.73 I^2^ = 69%), 10% citric acid (MD −236.48 [−579.92, 106.95] *p* = 0.18 I^2^ = 92%), etidronic acid (MD 9975.44 [−1580.53, 21,531.42] *p* = 0.09 I^2^ = 97%), and 0.008% BAC (MD −16.34 [−94.91, 62.24] *p* = 0.68) very low certainty, according to GRADE.

When compared to citric acid phosphate buffer, citrate buffer, and 1% phytic acid, samples irrigated with 17% EDTA presented higher TGF-β1 released levels (MD −215.33 [−284.63, −146.04] *p* < 0.00001 I^2^ = 94%); (MD −213 [−282.83, −143.94] *p* < 0.00001 I^2^ = 94%); and (MD −17,389.23 [−19,789.87, −14,988.59] *p* < 0.00001 I^2^ = 20%), respectively. However, samples irrigated with 10% EDTA (MD 159.83 [46.24, 273.41] *p* = 0.006 I^2^ = 0%), 1.5% NaOCl + 17% EDTA (MD 7421.77 [3393.21, 11,450.33] *p* < 0.0003 I^2^ = 96%), and 2.5% NaOCl + 17% EDTA (MD 34,655.65 [322,212.97, 37,098.34] *p* < 0.00001 I^2^ = 99%) presented higher TGF-β1 released levels than samples irrigated with 17% EDTA. The certainty of evidence ranged from very low to low.

#### 3.4.3. Detection of TGF-β1 by the Immunogold Method 

**Figure 4:**
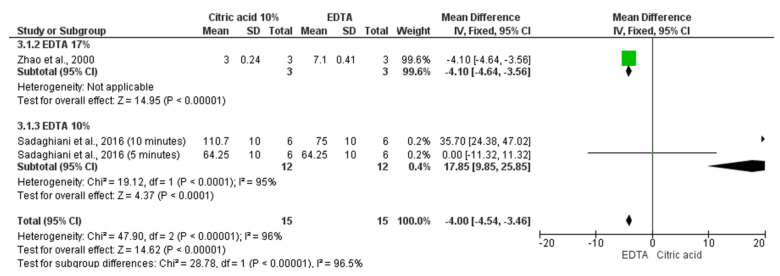
Forest plot for comparisons of TGF-β1 detected by the immunogold method after exposure to EDTA versus 10% citric acid.

Considering two studies [32,35], three comparisons of means and standard deviation of TGF-β1 detected by the immunogold method after exposure to EDTA versus 10% citric acid were included in this analysis, and a total of 30 samples was evaluated in this analysis (Figure 4). While 17% EDTA irrigation resulted in higher levels of TGF-β1 (MD −4.10 [−4.64, −3.56] *p* < 0.00001), 10% EDTA induced lower levels of TGF-β1 (MD 17.85 [9.85, 25.85] *p* < 0.0001 I^2^ = 95%), when both were compared to 10% citric acid. The level of evidence was considered as very low. 

## 4. Discussion

### 4.1. Regenerative Endodontics

For endodontic regeneration to occur, the conducive microenvironment needs to be disinfected, and site preparation becomes essential [36]. In this context, the chemical cleaning phase through the use of endodontic irrigants is emphasized [37], mainly in cases of immature teeth, since no instrumentation is preferable due to dentin fragility. Furthermore, studies have shown that the remaining layer of intact predentin may contain soluble proteins and growth factors to be potentially extracted that may act upon dental pulp stem cells [7,31]. 

In addition to the antimicrobial and decalcifying properties [38], irrigants may contribute to the release of dentin-growth factors [39], which are determinant molecules to promote the phenotypic differentiation of stem cells [40]. This systematic review and meta-analyses investigated the evidence on the impact of different endodontic irrigant solutions in releasing radicular dentin-growth factors.

Almost all included studies assessed the release of TGF-β1, a molecule recognized for its key role in cell recruitment and mobilization to provide a higher production of mineralized dentin [29]. The release of BMP-2 and BMP-7, known to play a significant function in odontoblastic differentiation and the induction of dentin sialophosphoprotein, was also identified for several different irrigant treatments [24,27,32]. Conversely, FGF and VEGF, which increase cellular proliferation and exert angiogenic effects in regenerative endodontics [10], were investigated in few studies [23,25,27,32,33]. Nevertheless, only TGF-β1 release results enabled comparisons through meta-analyses. Ideally, to provide broader evidence regarding irrigants’ influence, the release of distinct growth factors should be quantitatively compared in future studies. 

One of the challenges of endodontic regeneration is to recruit mesenchymal cells so that later, differentiation occurs in cells similar to odontoblasts [33]. Some studies have investigated the relationship between stem cells and the release of dentin-growth factors induced by endodontic irrigants. Interestingly, etidronic acid was more effective than EDTA both in the migration of stem cells and in the long-term release of growth factors, justifying the chemotactic ability of these molecules [29]. Similarly, the increased migration of stem cells and release of growth factors were observed after conditioning with citric acid solutions [28]. In addition, a greater migration of stem cells could be directly linked to higher amounts of growth factors detected in the root canal [33]. The positive results of migration tests with stem cells reinforce the dentin-growth factors’ functionality released into the root canal. 

Regarding cell adhesion, citric acid favored cell attachment [28], while both EDTA and citric acid have no adverse effects on cell proliferation and attachment to root dentin [27]. In differentiation assays, the conditioning with EDTA and citric acid seemed to trigger a positive regulation of mineralized differentiation genes. The cells had an odontoblastic morphology, with elongated bodies and long extracellular processes over the dentin surface [32]. Concerning cell viability, neither EDTA nor citric acid present significant cytotoxic effects compared to nonirrigated dentin [13], although citric acid resulted in higher cell counts as compared to EDTA [28].

### 4.2. Methodological Issues

Most of the included studies employed dentin slice models, whose preparation can be easily performed, allowing a standardized thickness and height for an accurate evaluation [29]. However, the use of these discs would imply an assessment of the release from the entire dentin content, including the inner and external surfaces, distinct from that measured from the inner surface of root canals by using root segments [13]. In this sense, the choice for the type of sample most probably affects the final result of the test, and it is possible to infer that the use of root segments would be closer to the clinical situation, where the action of the irrigant occurs only inside the root canal. In this regard, the current systematic review results point out that more studies with high methodological quality should be performed to clarify the influence of irrigants on the release of growth from radicular intracanal dentin.

Growth factors were mainly assessed in liquid samples by ELISA assays. If such samples were collected during irrigation, the growth factors detected could represent those lost by washing if the results were extrapolated to the clinical setting. By contrast, immunoassays evaluated the direct exposure of growth factor molecules on the dentin surface, which possibly could substantially influence stem cell behavior [10]. 

Furthermore, the ELISA tests are susceptible to acidic conditions. Changes in amino acids and the dissociation of binding proteins caused by protonation can block the target protein’s signal and create a competition for binding points with ELISA antibodies [28,41], contributing to an apparent underestimation of the release of growth factors by some irrigants [28,42]. Nevertheless, ELISA was the most common method used in the included studies, and it may still be suitable for this assessment, as long as such limitations can be mitigated. Alternatively, after using the irrigants, a buffering solution could be added, allowing the evaluation in this buffered solution [27] and sample collection after the irrigation procedure [13]. 

Another important methodological issue is the clinical relevance of the chosen experimental times. Prolonged exposure to demineralizing agents appears impractical in a clinical situation [10,32]. Scelza et al. [43] reported that 3 min was enough for the demineralization action of EDTA and citric acid, while a prolonged time of exposure to these substances can interfere with dentin’s structural properties [44].

### 4.3. The Action of Irrigants in the Release of Dentin-Growth Factors

Bearing in mind that irrigation procedures are strongly recommended during regenerative endodontic approaches, it is necessary to ensure that they do not neutralize bioactive molecules’ release [10] and, if possible, encourage their release. Regarding the irrigation protocols, sodium hypochlorite solutions are widely used in regenerative endodontics due to the intense antibacterial action and their ability to dissolve organic debris [45]. However, these solutions in high concentrations, such as 3%, were less effective in releasing dentin-growth factors, compared to 17% EDTA [35]. In this way, 1.5% hypochlorite irrigant solutions have been recommended in regenerative endodontics protocols [46]. Of note, since this substance may be cytotoxic to stem cells [47], EDTA has been used as a final irrigating solution to neutralize possible harmful effects [48]. Interestingly, NaOCL and EDTA irrigant solutions’ sequential use significantly favored the release of TGF-β1 [26,33]. 

Indeed, evidence of the extensive use of EDTA comes from the fact that all included studies used this type of irrigant solution in their comparisons. The meta-analyses from ELISA assessments showed that 10% EDTA induced significantly greater TGF-β1 release than 10% citric acid, corroborating with the results of three studies that showed a superior effect of EDTA compared to other solutions [30,31,34]. The better outcomes identified for EDTA may be related to its decisive demineralizing and solubilizing action on hard dental tissues, releasing TGF-β1 molecules trapped in the dentin [31]. However, 17% EDTA had similar effects to 10% citric acid and a lower release than 10% EDTA. A possible reason for this difference may come from the higher chelating capacity reported for 10% EDTA than 17% EDTA [49]. However, this last comparison in the meta-analysis (10% EDTA and 17% EDTA) should be considered with caution since it was based on a single study [10]. 

The meta-analysis also favored EDTA on the release of TGF-β1 when considering the immunogold assays. However, in one comparison, 10% citric acid significantly exposed more TGF-β1 than 10% EDTA, corroborating two ELISA assays studies included in the qualitative analysis reporting that citric acid had more pronounced effects on dentin-growth factors release when compared to 17% EDTA [27,28]. A possible reason for these results is related to a more acidic medium that may promote the denaturation of a peptide associated with the latent TGF-β complex, which in turn causes a higher release of TGF-β in its active form, leading to increased biological effects [13,50]. Such an idea is corroborated by the findings of Galler et al. [10], which showed that the action of an acidic medium before the use of EDTA could accelerate this release of growth factors.

Regarding the qualitative assessment of the release of growth factors other than TGF-β1, there is not sufficient evidence for the determination of differences for most of those, including FGF, for which no distinctions were detected between the tested solutions [27,33] and VEGF, which had decreased release reported after treatment with irrigants [27,32]. This result may be due to the low concentration of VEGF found in dentin along with its short half-life [51,52]. Citric acid was responsible for a more significant release of BMP-2 on a single ELISA evaluation, with no statistical difference when compared to EDTA in the immunogold assay in the same study [32]. The release of BMPs, VEGF, IGF-I, and FGF met no consensus between studies, and the low to very low certainty of the available evidence (Supplementary Materials Table S4) demands further studies to compare this essential protein release different irrigant treatments.

It is noteworthy that Hancerliogullari et al. [24] and Aksel et al. [26] showed a significant increase in the release of growth factors when the irrigating solution was agitated by an ultrasonic system or with the use of laser—a finding that corroborates the study by Widbiller et al. [53]. The dynamic movements generated by the action tend to result in well-debrided root canal walls, which seem to increase the release of growth factors.

The studies included in this review showed that irrigant substances significantly affect the release of growth factors. Among the studied irrigating solutions, citric acid and EDTA seem to have a higher effect. Both EDTA and citric acid are widely known for removing the smear layer [28]. However, an additional point to be considered is the clinical applicability of irrigant solutions, as studies show that EDTA could lead to dentin fragility, perhaps due to dentinal tissue damage, in contrast to the relatively safer use of citric acid [54,55,56].

Considering the overall outcomes, the results favored the use of EDTA according to the meta-analyses performed. However, while the comparisons identified statistical differences, only few studies were included, and high heterogeneity indexes were observed. Furthermore, although the comparisons did not present problems related to the risk of bias and publication bias, the dose-response gradient or association with the effects, severe problems of inconsistency, indirectness, and imprecision were detected. Therefore, the certainty of the evidence was rated as very low when applying an adapted GRADE assessment of the certainty of evidence. Therefore, such evidence may change according to the results and conclusions from future studies. 

It is important to notice that the studies in which EDTA fared better than citric acid did not elucidate how they minimized the ELISA test limitations, as discussed in the methodological issues section. Galler et al. [10] only stated that interferences from the test solutions in the ELISA measurements were excluded. Sadaghiani et al. [32], who made no mention of the limitations of ELISA in this model, obtained different results by immunoassays, with lower differences between EDTA and citric acid regarding TGF- β1 release. 

A limitation related to the meta-analyses was that some comparisons were based on a single or few studies with low statistical power and high heterogeneity. Due to the low number of studies, we were unable to isolate differences related to the type of sample (slices, root segments, or dentin powder), which could influence the amount of released growth factors detection. The choice for the GRADE approach may also pose a limitation, as it was designed for the evaluation of randomized clinical trials [21], and a considerable amount of adaptation and interpretation was required for its use with in vitro studies. While its use for animal studies has already been discussed [57], the present review would have benefitted from a validated adaptation of the GRADE framework for in vitro settings. Nevertheless, presenting the results of these analyses, rather than excluding them for these limitations, may refine the identification of gaps in the up-to-date body of knowledge. In this regard, the results point out that more studies with high methodological quality should be performed to clarify irrigants’ influence on the release of growth factors from radicular intracanal dentin.

The limitations in extrapolating results from in vitro settings to the clinical practice should be considered when reaching conclusions of their contribution to therapeutic decision making. Nevertheless, the difficulties in accessing such molecular issues in clinical research also point to the relevance of these in vitro studies as primary data sources, mainly when performed to the highest methodological rigor and quality. In this sense, the present review shows that in vitro studies indeed can identify differences in the pattern of dentin-growth factors release with the choice of endodontic irrigants. Therefore, the major limitation of the available scientific literature is the high heterogeneity, which would not be initially considered an inherent characteristic of in vitro studies. In fact, such a trait evidences the use of very different protocols performed with diverse methodologies, seeking to answer the same question. Thus, future actions on the standardization of these protocols, considering the main methodological issues already raised in this review, could lead to higher methodological quality necessary to achieve conclusive recommendations. Therefore, the authors recommend the proposal of standardized studies using analyzes both in root segments, which are closest to clinical situations, and subsequent analysis of dentin powder to measure the amount of growth factor remaining in dentin with the potential to be released. In addition, studies may resort to the use of irrigants at well-established times, such as the 5 min recommended by the American Association of Endodontists [46]. Hence, more than pointing out the best irrigant solution, the present work expects to bring attention to critical aspects that may help further in vitro studies to benefit clinical decision making on regenerative endodontics conclusively.

## 5. Conclusions

The overall assessment of studies included in this systematic review identified an increased release of TGF-β1 in dentin treated with EDTA. However, the high heterogeneity and the low to very low certainty of the available evidence demand further studies before recommending it as the best irrigant solution for attaining dentin-growth factors release in regenerative endodontics.

## Figures and Tables

**Table 1:** Main characteristics of the included studies.

Study (Year)	Type of Sample	Root Canal Irrigant	Growth Factors	Evaluation Method
Kucukkaya Eren et al., 2021 [22]	Dentin slices	17% EDTA and 17% EDTA + 0.008% benzalkonium chloride	TGF-β1	ELISA
Khan et al., 2021 [23]	Dentin slices and Root canal segment	17% EDTA, 9% etidronic acid, and 1% phytic acid	VEGF	ELISA
Hancerliogullari et al., 2021 [24]	Root canal segment	17% EDTA, 10% citric acid (break)Both irrigants was tested with follow irrigation activation technique (conventional syringe irrigation, passive ultrasonic irrigation, PUI, and Er:YAG laser activation	TGF-β1, IGF-I, BMP-7 and VEGF-A	ELISA
Ferreira et al., 2020 [25]	Root canal segment	2.5% NaOCl, 2% chlorhexidine, and 10% EDTA	TGF-β1 and VEGF	ELISA
Aksel et al., 2020 [26]	Dentin slices	1.5% NaOCl + PBS + 17% EDTA + PBS, 17% EDTA with Nanobubble water, 17% EDTA activated with ultrasonic, 17% EDTA with Nanobubble water activated with ultrasonic, and phosphate-buffered saline (PBS)	TGF-β	ELISA
Atesci et al., 2020 [27]	Dentin slices and Powdered dentine *	17% EDTA, 10% citric acid, 1% phytic acid, 37% phosphoric acid, distilled water	TGF-β1, BMP-2, FGF-2 and VEGF	ELISA
Ivica et al., 2019 [28]	Dentin slices	10% Citric Acid, 17% EDTA and phosphate-buffered saline	TGF-β1	Slot blot
Deniz Sungur et al., 2019 [29]	Dentin slices	17% EDTA, 1% phytic acid, 9% etidronic acid, and distilled water	TGF-β	ELISA
Chae et al., 2018 [13]	Root canal segment	Saline, 17% EDTA, 10% citric acid, 10% phosphoric acid, and 37% phosphoric acid	TGF-β1	ELISA
Duncan et al., 2017 [30]	Powdered dentine	10% EDTA, valproic acid, trichostatin A and suberoylanilide hydroxamic acid	TGF-β1	ELISA
Gonçalves et al., 2016 [31]	Dentin slices	EDTA 10%, NaOCl 2,5% and phosphate-buffered saline	TGF-β1	ELISA
Sadaghiani et al., 2016 [32]	Dentin slices	10% EDTA, 37% phosphoric acid, 10% citric acid, 25% polyacrylic acid, buffered saline, and calcium hydroxide	TGF-β1, BMP-2 and VEGF	ELISA and Immunogold method
Zeng et al., 2016 [33]	Root canal segment	1.5% NaOCl + 17% EDTA, 2.5% NaOCl + 17% EDTA, 17% EDTA, and deionized water	TGF-β1 and bFGF	ELISA
Galler et al., 2015 [10]	Dentin slices	10% EDTA, 17% EDTA, 10% citric acid, citrate buffer, and citric acid phosphate buffer	TGF-β1	ELISA
Graham et al., 2006 [34]	Powdered dentine	10% EDTA and calcium hydroxide	TGF-β1	ELISA
Zhao et al., 2000 [35]	Dentin slices	3% NaOCl, 17% EDTA, 10% citric acid, and phosphate-buffered saline	TGF-β1 TGF-β2 TGF-β3	Immunogold method

**Table 2:** Main conclusions of the included studies.

Study (Year)	Main Conclusions
Kucukkaya Eren et al., 2021 [22]	Both 17% EDTA and 17% EDTA + 0.008% benzalkonium chloride were similar in the amount of TGF-β1 released by dentin.
Khan et al., 2021 [23]	VEGF release by 9% etidronic acid was greater in dentin cylinders than 17% EDTA and 1% phytic acid, however similar between irrigants groups.in the dentin discs analysis.
Hancerliogullari et al., 2021 [24]	The 17% EDTA caused significantly more IGF-I release than 10% citric acid, while for TGF-β1, BMP-7, and VEGF-A, both irrigants were equally effective.
Ferreira et al., 2020 [25]	The 2% chlorhexidine and 10% EDTA irrigants released significantly more TGF- β1 than 2.5% NaOCl. No VEGF release was detected for any group.
Aksel et al., 2020 [26]	Although there is no significant difference between the groups of irrigants used, the ultrasonic activation enhanced the TGF- β release.
Atesci et al., 2020 [27]	For TGF- β1, 10% citric acid was responsible for releasing significantly more than EDTA, IP6, and with no statistically significant difference when compared to 37% phosphoric acid. For VEGF, there was a very minor release with no significant difference, while for BMP-2 and FGF-2, the release was similar to all irrigants.
Ivica et al., 2019 [28]	The 17% EDTA released a 5-fold higher concentration of TGF- β1 than 10% citric acid.
Deniz Sungur et al., 2019 [29]	The greatest release of TGF-β1 was obtained in the 9% etidronic acid group, whilethe lowest in the 1% phytic acid group, with no significant difference between groups.
Chae et al., 2018 [13]	The use of 10% citric acid released significantly more TGF- β1 than 17% EDTA and 10% phosphoric acid, while 37% phosphoric acid and saline released the least amount.
Ducan et al., 2017 [30]	The 10% EDTA promoted significantly greater release of TGF- β1 than valproic acid, trichostatin A, and suberoylanilide hydroxamic acid.
Gonçalves et al., 2016 [31]	The 10% EDTA released significantly more TGF- β1of dentin matrix than 2.5% NaOCl or PBS.
Sadaghiani et al., 2016 [32]	Under the immunogold method, calcium hydroxide significantly increased the release of TGF-β1 within 5 min of conditioning, BMP-2 and VEGF within 10 min. The 10% EDTA, 10% citric acid, and 37% phosphoric acid showed intermediate values for TGF-β1 release, while for BMP-2 and VEGF, the phosphoric acid was lower in the time of 10 min. In the ELISA method, only 17% EDTA detected TGF-β1 release, whereas for BMP-2 and VEGF the most effective irrigant was 10% citric acid.
Zeng et al., 2016 [33]	The groups with 1.5% NaOCl + 17% EDTA and 2.5% NaOCl + 17% EDTA had significantly higher release of TGF- β1 than 17% EDTA, with a peak release at day 1. The release of bFGF was detected at a low level in all irrigants.
Galler et al., 2015 [10]	Conditioning with 10% EDTA resulted in the release of the highest amounts of TGF- β1, while 17% EDTA was less effective. The release after treatment with citric acid and its variations was significantly smaller than EDTA.
Graham et al., 2006 [34]	The 10% EDTA released higher concentrations of TGF- β1 from dentin than calcium hydroxide.
Zhao et al., 2000 [35]	Conditioning with 17% EDTA generated a greater release of TGF-β1 while treatments with 10% citric acid and 3% NaOCl revealed smaller amounts of this isoform. TGF-β2 and -β3 isoforms could not be detected in samples with any of the irrigants.

**Table 3:** Final results of the quality assessment of the included studies, according to the SciRAP tool.

Study (Year)	Reporting Quality	Methodological Quality	Relevance	Quality Rating
Kucukkaya Eren et al., 2021 [22]	97.73	100	Directly relevant	Low risk
Khan et al., 2021 [23]	93.75	100	Directly relevant	Low risk
Hancerliogullari et al., 2021 [24]	90	91.67	Directly relevant	Low risk
Ferreira et al., 2020 [25]	93.75	91.67	Directly relevant	Low risk
Aksel et al., 2020 [26]	90.48	96.43	Directly relevant	Low risk
Atesci et al., 2019 [27]	83.33	92.31	Directly relevant	Low risk
Ivica et al., 2019 [28]	88.1	96.43	Directly relevant	Low risk
Sungur et al., 2019 [29]	97.62	100	Directly relevant	Low risk
Chae et al., 2018 [13]	78.57	78.57	Directly relevant	Low risk
Duncan et al., 2016 [30]	92.86	95.83	Directly relevant	Low risk
Gonçalves et al., 2016 [31]	90.48	92.31	Directly relevant	Low risk
Sadaghiani et al., 2016 [32]	88.1	92.31	Directly relevant	Low risk
Zeng et al., 2016 [33]	92.86	92.31	Directly relevant	Low risk
Galler et al., 2015 [10]	78.13	95.83	Directly relevant	Low risk
Graham et al., 2006 [34]	80.95	92.31	Directly relevant	Low risk
Zhao et al., 2000 [35]	71.88	79.17	Directly relevant	Low risk

## Data Availability

Data related to the complete quality assessment as well as Supplementary Tables S1–S4 are available from Mendeley Data: https://data.mendeley.com/datasets/s8znfrtjxx/1.

## Supplementary Materials

Supplementary materials are available at www.mdpi.com/article/10.3390/ma14195829/s1 and also on the Mendeley Data platform: https://data.mendeley.com/datasets/s8znfrtjxx/1. Table S1: electronic database used and search strategy; Table S2: time of exposure of samples to irrigants, assessment time, sample size, unit of measure, types of root canal irrigants solutions, mean and standard deviation of the concentration of TGF-β1 obtained, evaluation methods, and summary of the risk of bias assessment from studies included; Table S3. certainty of evidence in meta-analysis results; Table S4. quality certainty of evidence for FGF, VEGF, BMP2, IGF-I, BMP-7, and overall growth factors.
