# Supplementary material for: Effect of Different Root Canal Irrigant Solutions on the Release of Dentin-Growth Factors: A Systematic Review and Meta-Analysis
Source: Materials (Basel). 2021 Oct 5;14(19):5829. doi: 10.3390/ma14195829 (PMC8510123; doi:10.3390/ma14195829)
Supplement: Supplementary file 1 [file materials-14-05829-s001.zip › materials-1370123-supplementary.pdf]

Effect of root canal irrigants solutions on the release of dentin-growth factors: A systematic review and meta-analysis

Tavares et al.

Table S1. Electronic database used and search strategy.

Database	.Search Strategy
PubMed	**#1** ("root canal irrigants"[MeSH Terms] OR (root canal irrigant[Title/Abstract] OR root canal irrigants[Title/Abstract])) OR "sodium hypochlorite"[MeSH Terms]) OR Sodium hypochlorite[Title/Abstract]) OR "chlorhexidine"[MeSH Terms]) OR Chlorhexidine[Title/Abstract]) OR "edetic acid"[MeSH Terms]) OR Edetic Acid[Title/Abstract]) OR EDTA[Title/Abstract]) OR Ethylenedinitrilotetraacetic Acid[Title/Abstract]) OR "citric acid"[MeSH Terms]) OR Citric acid[Title/Abstract])<br><br>**#2** (Growth Factor[Title/Abstract]) OR "transforming growth factor beta1"[MeSH Terms]) OR Transforming Growth Factor beta1[Title/Abstract]) OR TGF-beta1[Title/Abstract]) OR TGF-beta-1[Title/Abstract]) OR TGF beta 1[Title/Abstract]) OR "transforming growth factor beta2"[MeSH Terms]) OR Transforming Growth Factor beta2[Title/Abstract]) OR TGF-beta2[Title/Abstract]) OR TGF-beta-2[Title/Abstract]) OR TGF beta 2[Title/Abstract]) OR "transforming growth factor beta3"[MeSH Terms]) OR Transforming Growth Factor beta3[Title/Abstract]) OR TGF-beta3[Title/Abstract]) OR TGF-beta-3[Title/Abstract]) OR TGF beta 3[Title/Abstract]) OR "bone morphogenetic proteins"[MeSH Terms]) OR Bone Morphogenetic Protein[Title/Abstract]) OR Bone Morphogenetic Proteins[Title/Abstract]) OR BMP[Title/Abstract]) OR "bone morphogenetic protein 2"[MeSH Terms]) OR Bone Morphogenetic Protein 2[Title/Abstract]) OR BMP2[Title/Abstract]) OR "bone morphogenetic protein 4"[MeSH Terms]) OR Bone Morphogenetic Protein 4[Title/Abstract]) OR BMP4[Title/Abstract]) OR "bone morphogenetic protein 7"[MeSH Terms]) OR Bone Morphogenetic Protein 7[Title/Abstract]) OR BMP7[Title/Abstract]) OR "insulin-like growth factor i"[MeSH Terms]) OR Insulin-Like Growth Factor I[Title/Abstract]) OR IGF-1[Title/Abstract]) OR IGF-I[Title/Abstract]) OR "insulin-like growth factor ii"[MeSH Terms]) OR Insulin-Like Growth Factor II[Title/Abstract]) OR IGF-2[Title/Abstract]) OR IGF-II[Title/Abstract]) OR "hepatocyte growth factor"[MeSH Terms]) OR Hepatocyte Growth Factor[Title/Abstract]) OR "vascular endothelial growth factors"[MeSH Terms]) OR Vascular Endothelial Growth Factor[Title/Abstract]) OR VEGFs[Title/Abstract]) OR "adrenomedullin"[MeSH Terms]) OR Adrenomedullin[Title/Abstract]))<br><br>**#3** ("tooth"[MeSH Terms] OR Tooth[Title/Abstract]) OR Teeth[Title/Abstract]) OR "dentin"[MeSH Terms]) OR Dentin[Title/Abstract]) OR Dentine[Transliterated Title]) OR Dentines[Title/Abstract]) OR "dentin, secondary"[MeSH Terms]) OR (("dentin"[MeSH Terms] OR "dentin"[All Fields]) AND Secondary[Title/Abstract])) OR Secondary Dentin[Title/Abstract]) OR Secondary Dentins[Title/Abstract]) OR "dental pulp cavity"[MeSH Terms]) OR Dental Pulp Cavity[Title/Abstract]) OR Pulp Chamber[Title/Abstract]) OR Pulp Chambers[Title/Abstract]) OR Pulp Canal[Title/Abstract]) OR Pulp Canals[Title/Abstract]) OR Root Canal[Title/Abstract]) OR Root Canals[Title/Abstract]) OR "dental enamel"[MeSH Terms]) OR Dental Enamel[Title/Abstract]) OR Dental Enamels[Title/Abstract]) OR Enamel[Title/Abstract]) OR Enamels[Title/Abstract])<br><br>**#1 AND #2 AND #3**
Scopus	**#1** ((TITLE-ABS-KEY (root AND canal AND irrigants) OR TITLE-ABS-KEY (sodium AND hypochlorite) OR TITLE-ABS-KEY (chlorhexidine) OR TITLE-ABS-KEY (edetic AND acid) OR TITLE-ABS-KEY (edta) OR TITLE-ABS-KEY (ethylenedinitrilotetraacetic AND acid) OR TITLE-ABS-KEY (citric AND acid)))<br><br>**#2** ((TITLE-ABS-KEY (growth AND factor) OR TITLE-ABS-KEY (transforming AND growth AND factor AND beta1) OR TITLE-ABS-KEY (tgf AND beta1) OR TITLE-ABS-KEY (transforming AND growth AND factor AND beta2) OR TITLE-ABS-KEY (tgf AND beta2) OR TITLE-ABS-KEY (transforming AND growth AND factor AND beta3) OR TITLE-ABS-KEY (tgf AND beta3) OR TITLE-ABS-KEY (bone AND morphogenetic AND proteins) OR TITLE-ABS-KEY (bmp) OR TITLE-ABS-KEY (bone AND morphogenetic AND protein 2) OR TITLE-ABS-KEY (bmp2) OR TITLE-ABS-KEY (bone AND morphogenetic AND protein 4) OR TITLE-ABS-KEY (bmp4) OR TITLE-ABS-KEY (bone AND morphogenetic AND protein 7) OR TITLE-ABS-KEY (bmp7) OR TITLE-ABS-KEY (insulin-like AND growth AND factor AND I) OR TITLE-ABS-KEY (igf-1) OR TITLE-ABS-KEY (igf-I) OR TITLE-ABS-KEY (insulin-like AND growth AND factor AND II) OR TITLE-ABS-KEY (igf-2) OR TITLE-ABS-KEY (igf-II) OR TITLE-ABS-KEY (hepatocyte AND growth AND factor) OR TITLE-ABS-KEY (vascular AND endothelial AND growth AND factor) OR TITLE-ABS-KEY (vegfs) OR TITLE-ABS-KEY (adrenomedullin)))<br><br>**#3** ((TITLE-ABS-KEY (tooth) OR TITLE-ABS-KEY (teeth) OR TITLE-ABS-KEY (dentin) OR TITLE-ABS-KEY (dentine) OR TITLE-ABS-KEY (secondary AND dentin) OR TITLE-ABS-KEY (dental AND pulp AND cavity) OR TITLE-ABS-KEY (pulp AND chamber) OR TITLE-ABS-KEY (pulp AND canal) OR TITLE-ABS-KEY (root AND canal) OR TITLE-ABS-KEY (dental AND enamel) OR TITLE-ABS-KEY (enamel)))<br><br>**#1 AND #2 AND #3**
Web of Science	**#1** TOPIC: (Root Canal Irrigants) OR TOPIC: (Root Canal Irrigant) OR TOPIC: (Sodium hypochlorite) OR TOPIC: (Chlorhexidine) OR TOPIC: (Edetic Acid) OR TOPIC:(EDTA) OR TOPIC: (Ethylenedinitrilotetraacetic Acid) OR TOPIC: (Citric acid)<br><br>**#2** TOPIC: (Growth Factor) OR TOPIC: (Transforming Growth Factor beta1) OR TOPIC: (TGF-beta1) OR TOPIC: (Transforming Growth Factor beta2) OR TOPIC: (TGF-beta2) OR TOPIC: (Transforming Growth Factor beta3) OR TOPIC: (TGF-beta3) OR TOPIC: (Bone Morphogenetic Proteins) OR TOPIC: (BMP) OR TOPIC: (Bone Morphogenetic Protein 2) OR TOPIC: (BMP2) OR TOPIC: (Bone Morphogenetic Protein 4) OR TOPIC: (BMP4) OR TOPIC: (Bone Morphogenetic Protein 7) ORTOPIC: (BMP7) OR TOPIC: (Insulin-Like Growth Factor I) OR TOPIC: (IGF-1) OR TOPIC: (IGF-I) OR TOPIC: (Insulin-Like Growth Factor II) OR TOPIC: (IGF-2)

	ORTOPIC: (IGF-II) OR TOPIC: (Hepatocyte Growth Factor) OR TOPIC: (Vascular Endothelial Growth Factor) OR TOPIC: (VEGFs) OR TOPIC: (Adrenomedullin)<br><br>**#3** TOPIC: (Tooth) OR TOPIC: (Teeth) OR TOPIC: (Dentin) OR TOPIC: (Dentine) OR TOPIC: (Secondary Dentin) OR TOPIC: (Dental Pulp Cavity) OR TOPIC: (Pulp Chamber) OR TOPIC: (Pulp Canal) OR TOPIC: (Root Canal) OR TOPIC: (Dental Enamel) OR TOPIC: (Enamel)<br>Indexes=CDerwent, EDerwent, MDerwent Timespan=All years<br><br>**#1 AND #2 AND #3**
**Virtual Health Library (Lilacs)**	tw:((tw:(root canal irrigants OR sodium hypochlorite OR chlorhexidine OR edetic acid OR edta OR ethylenedinitrilotetraacetic acid OR citric acid)) AND (tw:(growth factor OR transforming growth factor beta1)) AND (tw:(tooth OR teeth OR dentin OR dental pulp cavity OR root canal OR enamel)))

Table S2. Time of exposure of samples to irrigants, assessment time, sample size, unit of measure, types of root canal irrigants solutions, mean and standard deviation of the concentration of TGF-β1 obtained, evaluation methods and summary of the risk of bias assessment from studies included.

Studies included		Quantitative data extracted				Types of root canal irrigants solutions and Release of TGF-β1 (Means and standard deviations)																										Methodological aspects	
Study/ year		Exposure time	Assessment time	Sample size	TGF-β1 measurement unit assessed	PBS	H_2O	Saline	17% EDTA	10% EDTA	10% Citric Acid	2% chlorhexidine	10% Phosphoric acid	37% Phosphoric acid	2,5% NaOCl	3% NaOCl	Citrate buffer	Citric acid /phosphate buffer	$Ca(OH)_2$	Trichostatin A	Valproic acid	Suberoylanilide hydroxamic acid	1% Phytic acid	9% Etidronic acid	1.5% NaOCl + 17% EDTA	2.5% NaOCl + 17% EDTA	1.5% NaOCl + PBS + EDTA + PBS	EDTA with Nanobubble water	EDTA activated with ultrasonic 5 min	EDTA with Nanobubble water activated with	17% EDTA+0.008% BAC	Evaluation methods	Summary of Risk of Bias Assessment
¥Kucukkaya ya Eren et al. 2021 [1]	Disk with cells	1 min	1 min	n=9	pg/ml				763.8 ± 220.4																						**756.9** ±178.8	ELISA	Low risk
		5 min	5 min						740.8 ± 133.8																						**666.9** ±140.8		
		10 min	10 min						680.8 ± 226.2																						**634.6** ± 226.2		
	Disk without cells	1 min	1 min	n=9	pg/ml				590.8 ± 150																						**627.7** ± 144.2		
		5 min	5 min						600 ± 139,6																						600 ± 148.8		
		10 min	10 min						611.5 ± 145.4																						**521.5** ± 155.8		
Hancerliogullari et al. 2021 [2]		5 min	RST	n=30	pg/ml				29840 ± 8770		29950 ± 10390																					ELISA	Low risk
¥Ferreira et al. 2020 [3]		5 min	5 min	n=8	pg/ml					326 ± 33,8		345 ± 68,7			73 ± 25,4																	ELISA	Low risk
¥Aksel et al. 2020 [4]**		NaOCl 5 min PBS 3 min EDTA 5 min	24h	n=9	pg/ml	**728** ± 20																					**722** ± **107**	**772** ± 63	**872** ± 83	**799** ± 72		ELISA	Low risk
¥Atesci et al., 2020* [5]		5 min	5 min	n=6	pg/mg		98,86 ± 18		225,28 ± 39		494,29 ± 51			337,11 ± 51									281,99 ± 43									ELISA	Low risk

Ivica et al., 2019 [6]		10 min	10 min	n=3	ng per dentin disk	0			66 ± 3		382 ± 30																					ELISA	Low risk
¥Sungur et al., 2019 [7]**		5 min	4h	n=9	pg/ml		27000 ± 8000		36000 ± 4000														24000 ± 8000	21000 ± 7000								ELISA	Low risk
			1d	n=9	pg/ml		65000 ± 8000		70000 ± 4000														52000 ± 5000	62000 ± 5000									
			3d	n=9	pg/ml		97000 ± 7000		98000 ± 4000														77000 ± 8000	100000 ± 6000									
			5d	n=9	pg/ml		121000 ± 8000		123000 ± 2000														102000 ± 8000	134000 ± 4000									
			7d	n=9	pg/ml		151000 ± 3000		144000 ± 8000														129000 ± 1000	162000 ± 5000									
			14d	n=9	pg/ml		174000 ± 8000		172000 ± 8000														154000 ± 8000	200000 ± 7000									
			28d	n=9	pg/ml		196000 ± 8000		192000 ± 8000														176000 ± 8000	227000 ± 7000									
¥Chae et al., 2018 [8]		5 min	5 min	5 g	pg/ml			47 ± 7	231 ± 277		516 ± 302		240 ± 192	53 ± 12																		ELISA	Low risk
¥Ducan et al., 2016 [9]		14d	14d	n=9	pg/ml	29636 ± 1618			> 100000 ± 736											17272 ± 1324	11818 ± 736	27635 ± 736										ELISA	Low risk
Gonçalves et al., 2016 [10]	with predentin layer	1 min for EDTA and 5d for NaOCl	3d	n=30	pg/ml	MC				1262175 pg/ml					MC																	ELISA	Low risk
	without predentin layer		3d	n=30	pg/ml	MC				1197095 pg/ml					MC																		

¥Sadaghiani et al., 2016 (ELISA) [11]	5 min	5 min	n=6	pg/ml	MC				645 ± 48	MC			MC					MC													ELISA	Low risk
	10 min	10 min	n=6	pg/ml	MC				646 ± 48	MC			MC					MC														
¥Sadaghiani et al., 2016 (Immunogold) [11]	5 min	5 min	n=6	mean particle number	8,5 ± 2				12,85 ± 2	12,85 ± 2			15,95 ± 3					57,85 ± 7													Immunogold	
	10 min	10 min	n=6	mean particle number	12,14 ± 2				15 ± 2	22,14 ± 2			21,66 ± 3					43,81 ± 6														
¥Zeng et al., 2016 [12]	NaOCl (20 mL/5 min) + EDTA (20 mL/5 min)	4h	n=12	pg/ml		MC		8000 ± 3000																16000 ± 9000	29000 ± 9000						ELISA	Low risk
		1d	n=12	pg/ml		MC		6920 ± 4490																69040 ± 30410	59260 ± 3370							
		3d	n=12	pg/ml		MC		16250 ± 9560																15160 ± 6400	13040 ± 1050							
¥Galler et al., 2015 [13]	5 min	5 min	n=9	pg/ml				139 ± 117	327 ± 158	22 ± 9						9 ± 10	7 ± 6														ELISA	Low risk
	10 min	10 min	n=9	pg/ml				398 ± 344	427 ± 362	56 ± 45						9 ± 13	7 ± 6															
	20 min	20 min	n=9	pg/ml				827 ± 363	923 ± 425	57 ± 80						9 ± 26	7 ± 6															
Graham et al., 2006 [14]	14d	14d	3 mg	pg/ml				1395 ± 36										364 ± 12													ELISA	Low risk
Zhao et al., 2000 [15]	1 min	2d	n-3	mean SEM / 100 mm2 from 10 random fields	1.3 ± 0.14			7.1 ± 0.41		3.0 ± 0.24					3.7 ± 0.28																Immunogold	Low risk

* The exposure time to phosphoric acid was 30s. ** The authors do not delimit which TGF-β isoform was measured. ¥Studies that had data extrapolated from graphs.
CSI: conventional syringe irrigation; PUI: passive ultrasonic irrigation; Er:YAG: Er:YAG laser activation with PIPS tip; RST: regardless of the sampling time; BAC: Benzalkonium chloride.

Table S3. Certainty of evidence in meta-analysis results.

Certainty assessment							Summary of findings		
							Study event rates (%)		Anticipated absolute effects
Samples (comparisons)	Risk of bias	Inconsistency	Indirectness	Imprecision	Other considerations	Overall certainty of evidence	With Other irrigants	With EDTA	Risk difference with EDTA
**EDTA 10% *versus* Citric acid 10%**									
138 (6 comparisons)	not serious	serious [a]	serious [b,c]	serious [d]	strong association dose response gradient	⊕◯◯◯ VERY LOW	69	69	MD **555.63 lower** (671.54 lower to 439.72 lower)
**EDTA 17% *versus* distilled water**									
126 (7 comparisons)	not serious	serious [a]	serious [b,c]	very serious [d,e]	very strong association dose response gradient	⊕◯◯◯ VERY LOW	63	63	MD **714.52 lower** (4800.12 lower to 3371.08 higher)
**EDTA 17% *versus* EDTA 10%**									
54 (3 comparisons)	not serious	not serious	serious [b,c]	serious [a]	dose response gradient	⊕◯◯◯ VERY LOW	27	27	MD **159.83 higher** (46.24 higher to 273.41 higher)
**EDTA 17% *versus* Citric acid 10%**									
72 (4 comparisons)	not serious	very serious [a,f]	serious [b]	very serious [d]	strong association dose response gradient	⊕◯◯◯ VERY LOW	36	36	MD **236.08 lower** (575.93 lower to 103.77 higher)
**EDTA 17% *versus* Citric acid phosphate buffer**									
54 (3 comparisons)	not serious	serious [a]	serious [b,c]	very serious [d,e]	dose response gradient	⊕◯◯◯ VERY LOW	27	27	MD **215.33 lower** (284.63 lower to 146.04 lower)
**EDTA 17%** versus **Citrate buffer**									
54 (3 comparisons)	not serious	serious [a]	serious [b,c]	very serious [d,e]	dose response gradient	⊕◯◯◯ VERY LOW	27	27	MD **213.38 lower** (282.83 lower to 143.94 lower)
**EDTA 17%** versus **Phytic acid 1%**									

Table S3. Certainty of evidence in meta-analysis results.

Certainty assessment							Summary of findings		
126<br>(7 comparisons)	not serious	not serious	serious [b,c]	serious [d]	dose response gradient	⊕◯◯◯<br>VERY LOW	63	63	MD **17389.23 lower**<br>(19789.87 lower to 14988.59 lower)
**EDTA 17%** versus **Etidronic acid**									
123<br>(7 comparisons)	not serious	very serious [a,f]	serious [b,c,g]	very serious [d,e]	dose response gradient	⊕◯◯◯<br>VERY LOW	63	60	MD **9975.44 higher**<br>(1580.53 lower to 21531.42 higher)
**EDTA 17%** versus **NaOCl 1.5% + EDTA 17%**									
72<br>(3 comparisons)	not serious	serious [a]	serious [b,h]	very serious [d,e]	strong association<br>dose response gradient	⊕◯◯◯<br>VERY LOW	36	36	MD **7421.77 higher**<br>(3393.21 higher to 11450.33 higher)
**EDTA 17% *versus* NaOCl 2.5% + EDTA 17%**									
72<br>(3 comparisons)	not serious	serious [a]	serious [b,h]	serious [d]	very strong association<br>dose response gradient	⊕⊕◯◯<br>LOW	36	36	MD **34655.65 higher**<br>(32212.97 higher to 37098.34 higher)
**EDTA 17% versus 0.008% bac**									
54<br>(3 comparisons)	not serious	serious [a]	serious [b,h]	serious [d]	very strong association	⊕◯◯◯<br>VERY LOW	27	27	MD **16.34 lower**<br>(94.91 lower to 62.24 higher)
**Imunogold analysis**									
30<br>(3 comparisons)	not serious	very serious [a,f]	serious [b,c]	serious [d]	dose response gradient	⊕◯◯◯<br>VERY LOW	15	15	MD **4 lower**<br>(4.54 lower to 3.46 lower)

CI: Confidence interval; **MD:** Mean difference

a. Considerable and significant heterogeneity.

b. Included comparisons did not evaluate powdered dentin specims.

c. Included comparisons did not evaluate in root segments.

d. Total number of specims is less than 400.

e. Upper or lower confidence limit was greater than 25% of estimated effect.

f. Moderate variation in the effect estimates across comparations and little overlap of confidence intervals associated with the effect estimates.

g. Results should be restricted to etidronic acid 9%

h. Included comparisons did not evaluated dentin slices.

Table S4. Quality certainty of evidence for FGF, VEGF, BMP2, IGF-I, BMP-7 and overall growth factors

Certainty assessment							Certainty	Explanations
№ of studies	Study design	Risk of bias	Inconsistency	Indirectness	Imprecision	Other considerations		
**bFGF**								
1	In vitro studiy	not serious	not serious	serious [a]	serious [b]	all plausible residual confounding would suggest spurious effect, while no effect was observed<br>dose response gradient	⨁⨁◯◯<br>LOW	a. Analysis did not included dentin slices specims.<br>b. Total number of specims is lower than 400.
**VEGF**								
5	In vitro studies	not serious	very serious [c]	not serious	serious [b]	all plausible residual confounding would suggest spurious effect, while no effect was observed<br>dose response gradient	⨁◯◯◯<br>VERY LOW	b. Total number of specims is lower than 400.<br>c. Sadaghiani et al. report superiority of citric acid, Atesci et al, Khan et al. and Hancerliogullari et al. reported similarity between groups, while Ferreira et al. 2020 did not detected VEGF.
**BMP-2**								

Certainty assessment							Certainty	Explanations
№ of studies	Study design	Risk of bias	Inconsistency	Indirectness	Imprecision	Other considerations		
2	In vitro study	not serious	serious [c]	serious [d]	very serious [b,e]	all plausible residual confounding would suggest spurious effect, while no effect was observed<br>dose response gradient	⊕◯◯◯<br>VERY LOW	b. Total number of specims is lower than 400.<br>c. Sadaghiani et al. report superiority of citric acid, while Atesci et al. report similarity between groups.<br>d. Analysis did not included root canal segments specims.<br>e. Upper and lower confidence estimated interval are greater than 25% of estimated effect.
**IGF-I**								
1	In vitro study	not serious	not serious	serious [f]	serious [b]	all plausible residual confounding would suggest spurious effect, while no effect was observed<br>dose response gradient	⊕⊕◯◯<br>LOW	f. Analysis did not included dentin slices and powdered dentine
**BMP-7**								
1	In vitro study	not serious	not serious	serious [f]	serious [b]	all plausible residual confounding would suggest spurious effect, while no effect was observed<br>dose response gradient	⊕⊕◯◯<br>LOW	f. Analysis did not included dentin slices and powdered dentine
**Overall growth factors**								
16	In vitro studies	not serious	very serious [g,h]	not serious	serious [b]	all plausible residual confounding would suggest spurious effect, while no effect was observed<br>dose response gradient	⊕◯◯◯<br>VERY LOW	b. Total number of specims is lower than 400.<br>g. Probably, considerable heterogeneity.<br>h. Estimated effect varied according growth factor and irrigant evaluated.

1. Kucukkaya Eren, S.; Bahador Zırh, E.; Zeybek, N.D.; Askerbeyli Örs, S.; Aksel, H.; Parashos, P. Effect of benzalkonium chloride addition to EDTA on attachment and proliferation of dental pulp stem cells on dentin and on transforming growth factor-β1 release. *Odontology* **2021**, *109*, 313-320, doi:10.1007/s10266-020-00545-5.
2. Hancerliogullari, D.; Erdemir, A.; Kisa, U. The effect of different irrigation solutions and activation techniques on the expression of growth factors from dentine of extracted premolar teeth. *International endodontic journal* **2021**, doi:10.1111/iej.13589.
3. Ferreira, L.N.; Puppin-Rontani, R.M.; Pascon, F.M. Effect of Intracanal Medicaments and Irrigants on the Release of Transforming Growth Factor Beta 1 and Vascular Endothelial Growth Factor from Cervical Root Dentin. *Journal of endodontics* **2020**, doi:10.1016/j.joen.2020.07.034.
4. Aksel, H.; Albanyan, H.; Bosaid, F.; Azim, A.A. Dentin Conditioning Protocol for Regenerative Endodontic Procedures. *Journal of endodontics* **2020**, *46*, 1099-1104, doi:10.1016/j.joen.2020.05.010.
5. Atesci, A.A.; Avci, C.B.; Tuglu, M.I.; Ozates Ay, N.P.; Eronat, A.C. Effect of Different Dentin Conditioning Agents on Growth Factor Release, Mesenchymal Stem Cell Attachment and Morphology. *Journal of endodontics* **2020**, *46*, 200-208, doi:10.1016/j.joen.2019.10.033.
6. Ivica, A.; Zehnder, M.; Mateos, J.M.; Ghayor, C.; Weber, F.E. Biomimetic Conditioning of Human Dentin Using Citric Acid. *Journal of endodontics* **2019**, *45*, 45-50, doi:10.1016/j.joen.2018.09.015.
7. Deniz Sungur, D.; Aksel, H.; Ozturk, S.; Yılmaz, Z.; Ulubayram, K. Effect of dentine conditioning with phytic acid or etidronic acid on growth factor release, dental pulp stem cell migration and viability. *International endodontic journal* **2019**, *52*, 838-846, doi:10.1111/iej.13066.
8. Chae, Y.; Yang, M.; Kim, J. Release of TGF-β1 into root canals with various final irrigants in regenerative endodontics: an in vitro analysis. *International endodontic journal* **2018**, *51*, 1389-1397, doi:10.1111/iej.12951.
9. Duncan, H.F.; Smith, A.J.; Fleming, G.J.; Reid, C.; Smith, G.; Cooper, P.R. Release of bio-active dentine extracellular matrix components by histone deacetylase inhibitors (HDACi). *International endodontic journal* **2017**, *50*, 24-38, doi:10.1111/iej.12588.
10. Gonçalves, L.F.; Fernandes, A.P.; Cosme-Silva, L.; Colombo, F.A.; Martins, N.S.; Oliveira, T.M.; Araujo, T.H.; Sakai, V.T. Effect of EDTA on TGF-β1 released from the dentin matrix and its influence on dental pulp stem cell migration. *Brazilian oral research* **2016**, *30*, e131, doi:10.1590/1807-3107BOR-2016.vol30.0131.
11. Sadaghiani, L.; Gleeson, H.B.; Youde, S.; Waddington, R.J.; Lynch, C.D.; Sloan, A.J. Growth Factor Liberation and DPSC Response Following Dentine Conditioning. *Journal of dental research* **2016**, *95*, 1298-1307, doi:10.1177/0022034516653568.
12. Zeng, Q.; Nguyen, S.; Zhang, H.; Chebrolu, H.P.; Alzebdeh, D.; Badi, M.A.; Kim, J.R.; Ling, J.; Yang, M. Release of Growth Factors into Root Canal by Irrigations in Regenerative Endodontics. *Journal of endodontics* **2016**, *42*, 1760-1766, doi:10.1016/j.joen.2016.04.029.
13. Galler, K.M.; Buchalla, W.; Hiller, K.A.; Federlin, M.; Eidt, A.; Schiefersteiner, M.; Schmalz, G. Influence of root canal disinfectants on growth factor release from dentin. *Journal of endodontics* **2015**, *41*, 363-368, doi:10.1016/j.joen.2014.11.021.
14. Graham, L.; Cooper, P.R.; Cassidy, N.; Nor, J.E.; Sloan, A.J.; Smith, A.J. The effect of calcium hydroxide on solubilisation of bio-active dentine matrix components. *Biomaterials* **2006**, *27*, 2865-2873, doi:10.1016/j.biomaterials.2005.12.020.
15. Zhao, S.; Sloan, A.J.; Murray, P.E.; Lumley, P.J.; Smith, A.J. Ultrastructural localisation of TGF-beta exposure in dentine by chemical treatment. *The Histochemical journal* **2000**, *32*, 489-494, doi:10.1023/a:1004100518245.
